# Epidemiology and Determinants of Vitamin D Deficiency in Eastern Nepal: A Community-Based, Cross-Sectional Study

**DOI:** 10.1155/2022/1063163

**Published:** 2022-09-09

**Authors:** O. Sherchand, J. K. Baranwal, B. Gelal

**Affiliations:** ^1^Department of Biochemistry, Nepal Medical College and Teaching Hospital, Kathmandu, Nepal; ^2^Department of Biochemistry, B.P. Koirala Institute of Health Sciences, Dharan, Nepal

## Abstract

**Objective:**

To estimate the prevalence of vitamin D deficiency in the eastern part of Nepal and identify the sociodemographic factors associated with it.

**Methods:**

A descriptive cross-sectional study was carried out among 324 participants between the ages 18 and 65 years from the Sunsari and Morang districts of Nepal. A semi-structured questionnaire helped obtain sociodemographic data followed by anthropometric measurements and blood sampling. 25(OH)D level was measured by chemiluminescence immunoassay (CLIA) via a fully automated Maglumi 1000 analyzer (SNIBE Co., Ltd., China). Serum 25(OH)D was classified as deficient, insufficient, and sufficient (<20 ng/ml, 20–29 ng/ml, and 30–100 ng/ml, respectively). The chi-square test was used to analyze the sociodemographic variables followed by a post hoc analysis. Significant variables were subjected to multivariate logistic regression.

**Result:**

181(55.9%) of the study population had vitamin D deficiency. There was significant association between vitamin D status and time of maximum sun exposure (chi square test = 11.1, *p* = 0.02), duration of sun exposure (chi-square test = 15.1, *p* = 0.004), type of meat intake (Fisher's exact test is 16.4, *p* = 0.01), frequency of fish intake (Fisher's exact test is 19.3, *p* = 0.001), and frequency of dairy intake (chi-square test=11.2, *p* = 0.02). In multivariate regression, consumption of dairy products ≥3/week had lower OR (95% CI) (0.3(0.1–0.8) *p* : 0.02) and weekly fish consumption had lower OR (95% CI) (0.06(0.008–0.6) *p* : 0.01) for vitamin D deficiency.

**Conclusion:**

The prevalence of vitamin D deficiency was relatively high in eastern Nepal. This highlights the need to create public awareness regarding the importance of bare skin sun exposure, nutritional sources of vitamin D, and the need to implement food fortification policies by the government.

## 1. Background

In recent years, hospitals across Nepal have witnessed an upsurge in vitamin D deficiency cases. Hospital-based studies conducted in western and southern Nepal depicted a high prevalence of vitamin D deficiency. [1, 2] However, data from the eastern parts are lacking which has warranted the need to determine the prevalence of vitamin D deficiency in this region.

Vitamin D deficiency is a pandemic health problem with multiple adverse health consequences. [3–5] Vitamin D is synthesized in the skin when exposed to ultraviolet (UV) rays from the sun. [5] Factors compromising the intensity of UV rays such as air pollution, latitude of residence, and skin pigmentation can impact vitamin D synthesis. [6–8] Another important source of vitamin D is dietary sources such as fish, cod liver oil, egg yolk, and mushroom, but the affordability of these items is beyond the reach of the majority of the households in Nepal [9, 10].

The data from India and China, the neighboring countries of Nepal, report a high prevalence of vitamin D deficiency [11,12]. However, there is a scarcity of data from Nepal as stipulated by a meta-analysis examining the prevalence of vitamin D deficiency in South Asians, which uncovered only two studies from Nepal. [13] Moreover, studies done in Nepal are hospital-based which can be subjected to clinical conditions that affect vitamin D metabolism, thus not representative of the community.

In this context, we conducted a community-based study to determine the prevalence of vitamin D deficiency and examined the different facets of vitamin D deficiency in terms of demographics, socioeconomic status, sun exposure factors, dietary, and lifestyle pattern.

## 2. Methods

### 2.1. Study Design, Site, and Participants

We conducted a community-based cross-sectional study in Sunsari and Morang districts (26˚N) of eastern Nepal, between the months of April 2019 and March 2020. The sample size was calculated using the following formula:

(1) $$\begin{array}{c} n=\frac{z^{2}pq}{d^{2}}, \end{array}$$

where *n*=sample size and *z* = *z* score at 95% confidence interval (1.96). *p* = estimated prevalence of vitamin D deficiency as 73.68% [2]. *q* = 1−p, *d* = margin of error (0.05), and *n* = 296.

We selected Belbari municipality, a major suburb of Morang district, and Dharan and Itahari, submetropolitan cities from Sunsari. With the aid of a community health worker, we recruited 337 participants, 225 from Morang and 112 from Sunsari. People were explained the purpose of our visit, and those giving written informed consent were enrolled. We excluded pregnant women, lactating mothers, and people taking vitamin D supplements, suffering from skin, liver, and kidney diseases. Following the exclusion criteria, 10 participants from Sunsari and 3 from Morang were excluded. This study was initiated after receiving ethical approval from the Institutional Review Committee, B.P. Koirala Institute of Health Sciences.

### 2.2. Demographic Variables

We administered a semi-structured questionnaire to obtain demographic data (age: young (18–44 years) and middle-aged and elderly (45–65 years), gender (male and female), ethnicity (Brahmin and Chettri, Newar, Janajati, and occupational caste), highest education level attained (up to primary school, intermediate, high school, and above high school), and occupation (professional and semiprofessional, skilled and semiskilled work, arithmetic skilled jobs, unskilled work, and unemployed)). An aggregate of educational status, occupation of the head of the household, and monthly income of the household helped determine socioeconomic status [14]. Smoking status was classified as current smoker, former, or never smoker. Alcohol intake was divided into drinks alcoholic beverages and does not drink. The questionnaire regarding indicators of sun exposure included duration of sun exposure (<15 minutes, 15–30 minutes, and >30 minutes), time of the day during maximum sun exposure (early morning: 6–8:59 am, late morning: 9–11:59 am, and afternoon: 12 noon to evening), skin colour (fair, light brown, and dark brown), and sunscreen use (uses SPF 15 and above and does not use). Physical activity was classified as active if they exercised >30 minutes/day at least 5 days a week, moderate if they exercised but lesser than the first criteria, and sedentary if they had no physical activity or irregular activity. Body mass index (BMI) was calculated as weight by height squared (kg/meter^2^) and classified as normal weight (18.5–22.9 kg/m^2^), overweight (≥23.0–24.99 kg/m^2^), and obese (≥25 kg/m^2^)] [15].

### 2.3. Dietary Pattern

The participants were asked if they followed a vegetarian or nonvegetarian diet. If nonvegetarian, the type of meat consumed was considered: (a) only chicken, (b) chicken and mutton, (c) chicken and pork, or (d) none. Food frequency was enquired by asking how often they consumed (1) meat ((a) ≥2/week, (b) once a week, (c) 2–3 times/month, or (d) none), (2) milk ((a) ≥3/week, (b) 1–2/week, (c) less than once a week), (3) dairy products as curd, cheese, butter, ghee, including egg ((a) ≥3/week, (b) 1–2/week, or (c) less than once a week), and (4) fish ((a) monthly, (b) weekly, or (c) rarely/never).

### 2.4. Serum 25(OH)D Measurement

1,25(OH)D is the active form of vitamin D; however, studies have identified serum 25(OH)D as the best marker of vitamin D status [16]. We used the same to find the vitamin D status. Venous blood samples were collected in a 3 ml plain vial and centrifuged to separate the serum. Aliquots of serum samples were transported to the biochemistry laboratory of BPKIHS maintaining a cold chain and stored at −20°C for subsequent batch analysis of serum 25(OH)D. We measured serum 25(OH)D with a Maglumi 1000 analyzer with chemiluminescence immunoassay (SNIBE Co., Ltd., China). The quality was assured using internal quality control provided by the manufacturer. Based on serum 25(OH)D, vitamin D status was classified as deficient: <20 ng/ml, insufficient: 20–29 ng/ml, and sufficient: 30–100 ng/ml. [16].

## 3. Statistical Analysis

The data were analyzed using Statistical Package of Social Sciences (SPSS) version 11.5 (SPSS Inc., Chicago, USA). We used descriptive statistics to express the baseline variables of the study. Prevalence of vitamin D status across categories of demographic, anthropometric variables, and dietary and lifestyle habits were compared using chi-square test. A *p* value of less than 0.05 was considered statistically significant at 95% confidence interval. Variables found significant in chi-square test were further subjected to post hoc test using Bonferroni corrected *p* value. The variables found to be statistically significant were then analyzed by multivariate regression using vitamin D status as the dependent variable.

## 4. Results

### 4.1. Baseline Characteristics

Out of 324 participants, 181 (55.9%) were female, 102 (31.5%) were from Sunsari, and 222 (68.5%) were from Morang. The majority (42%) of the participants were Brahman and Chhetri. 169 (52.2%) were middle-aged and elderly, and 96 (29.6%) were obese. 164 (50.6%) led a sedentary lifestyle. The time of maximum sun exposure for the majority of 167 (51.5%) was during the late morning. 175 (54%) received less than 15 minutes of sun exposure (Table 1). 305 (94.1%) consumed a nonvegetarian diet. 123 (38%) consumed mostly chicken. 147 (45.4%) consumed meat once a week. A majority of 199 (61.4%) consumed fish at monthly intervals. 168 (51.9%) drank milk less than once a week. 118 (36.4%) consumed dairy products 1–2 times/week (Table 2).

### 4.2. Serum 25(OH)D Status

**Figure 1:**
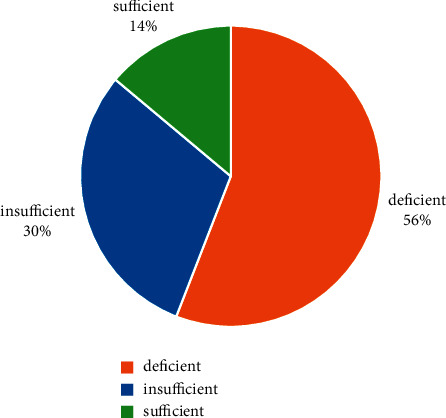
Pie chart showing the distribution of vitamin D status in Eastern Nepal.

Overall, 181 (55.9%) were vitamin D deficient, 98 (30.2%) were insufficient, and 45 (13.9%) had sufficient levels (Figure 1). There was a significant association between vitamin D status and gender (chi-square test = 6.2, *p* = 0.04) and education (chi-square test = 14.4, *p* = 0.02). However, after post hoc analysis, gender and education did not show significant association with vitamin D. There was significant association between vitamin D status and time of maximum sun exposure (chi-square test = 11.1, *p* = 0.02), duration of sun exposure (chi-square test = 15.1, *p* = 0.004), and skin colour (chi-square test = 17.9,*p* = 0.001). The post hoc test showed a significantly lower prevalence of vitamin D deficiency among people exposed to sun between 15 and 30 minutes on an average per day, while getting exposed for >30 minutes per day had a significantly higher prevalence of deficiency. Similarly, the prevalence of vitamin D sufficiency was significantly higher among people exposed to the early morning sun. Among skin colours, people with dark skin tones had a significantly higher prevalence of vitamin D deficiency than any other skin colour (Table 1).

Among dietary patterns, we found a significant association between vitamin D status and type of meat intake (Fisher's exact test is 16.4 and *p* = 0.01), frequency of fish intake (Fisher's exact test is 19.3, *p* = 0.001), and frequency of dairy intake (chi-square test=11.2, *p* = 0.02) (Table 2). Post hoc analysis revealed eating chicken was significantly associated with a lower prevalence of vitamin D deficiency, and such a relationship was not established with other types of meat. The frequency of fish consumption also showed significant results; people consuming fish weekly had a significantly higher prevalence of vitamin D sufficiency, while those who rarely or never consumed fish had a significantly higher prevalence of deficiency. People consuming dairy products less than once a week had a significantly higher prevalence of vitamin D deficiency.

These characteristics found to be significant on the post hoc test were further subjected to multivariate logistic regression analysis. The OR (95% CI) for vitamin D deficient and insufficient was compared against the reference vitamin D sufficient. Weekly fish consumption was a significant protective factor with crude OR (95% CI): 0.07(0.009–0.5) *p* : 0.01 and adjusted OR 0.06 (0.008–0.6) *p* : 0.01. Consumption of dairy products ≥3/week was also seen as a protective factor against vitamin D deficiency with OR (95% CI): 0.2(0.1–0.6) *p* : 0.005, and when adjusted with other factors, OR was 0.3(0.1–0.8) *p* : 0.02. People exposed to the sun for 15–30 minutes per day had lesser odds of vitamin D deficiency than those exposed to lesser duration OR 0.3(0.1–0.7) *p* : 0.004. However, adjusted odds were not significant (Table 3).

## 5. Discussion

Sunsari and Morang are terai (flat lands) districts of eastern Nepal with a lower tropical climate. [17] These regions experience abundant sunshine throughout the year. Despite sunny weather; we found a high prevalence of vitamin D deficiency. We tried to explore different aspects of sun exposure and determine the time of maximum sun exposure and duration of sun exposure needed for optimum vitamin D in this part of the world.

We found people who were exposed to early morning sunlight between 6 am and 8 : 59 am had a significantly greater percentage of sufficient vitamin D levels than those exposed to the sun later during the day. This finding contradicts the established notion that sun exposure between 10 am and 3 pm is the best for optimum levels of vitamin D. [5] This may be because most of our participants who were exposed to the sun later during the day claimed they used an umbrella to shield the hot sun and mostly chose to walk in shady areas while others stated they used transport means to get from one place to another, thus decreasing their duration in the sun.

In our study, we determined the optimum duration of sun exposure for adequate vitamin D levels was 15–30 minutes/day. The magnitude of solar radiation reaching the earth's surface is affected by latitude, seasons, and aerosols. [18] Hence, our finding may be relevant for our latitude, altitude, and seasons of sample collection which were autumn and spring.

We explored if vitamin D status varied between the ethnic groups present in eastern Nepal but found no significant variation. The prevalence of vitamin D deficiency was also comparable amongst the age groups and genders. This may be attributed to the increasing number of women labour force in Nepal venturing outside the confines of the house, thus increasing their chances of sun exposure. [19] The gap between the gender further narrows as beauty products and sunscreens are increasingly been marketed to men, thus giving both genders equal chances with the sun both in terms of sun exposure and sun barrier. [20].

We found a higher prevalence of vitamin D deficiency among people with dark brown skin. Dark-skinned people have larger amounts of melanin pigment which absorbs the ultraviolet rays of the sun and decreases the quantity available for the conversion of 7-dehydrocholesterol to previtamin D3. [8] This suggests that dark-skinned people would require a greater duration of sun exposure to synthesize a sufficient quantity of vitamin D. A study conducted in Northern Europe found insufficient dietary vitamin D and dark skin were the major risk factors for vitamin D insufficiency. [21] Similarly, a longitudinal study of parents and children found fairer-skinned children had higher levels of vitamin D. [22].

Dietary sources account for 5 to 10% of the total vitamin D and become crucial where there is inadequate sunlight exposure. [23] Major dietary sources of vitamin D include fish, fish liver oils, beef, pork, chicken, turkey, and eggs. [9] In our study, we analyzed dietary pattern as vegetarian versus nonvegetarian, type of meat consumed, frequency of meat, fish, milk, and dairy product consumption. We found participants who consumed fish on weekly basis had significantly higher number of vitamin D sufficient level. Indeed, fish is a natural source of vitamin D proven empirically by a meta-analysis of randomized controlled trial. According to this study, consuming ≥2 fish meals/week for at least 4 weeks significantly increased the 25(OH)D level. [24] We found people who consumed ≥3times/week dairy products had significantly lower prevalence of vitamin D deficiency. A study conducted to determine the predictors of vitamin D status by Levy et al. found fortified food, dairy, and vitamin D supplement consumption were positive predictors of the vitamin D status. [25] Indeed, dairy products as butter and cheese contain up to 10 *μ*g/kg of vitamin D due to its high fat content. [26].

This study has its strengths and limitations. This study helps explore factors determining the vitamin D status in a region of eastern Nepal. It takes into account the various sociocultural factors as ethnic diversity, occupation, and dietary habits that are unique to that region of Nepal. However, this paper may also be subjected to recall bias as participants answered the questionnaire form based on their recollection. Vitamin D was not measured by HPLC-MS/MS which is considered as reference procedure by the international organizations [27]. Hence, our finding may be subject to bias. Furthermore, our study was a cross-sectional one, as a result we cannot establish temporal trajectories.

## 6. Conclusion

The prevalence of vitamin D deficiency was relatively high in eastern Nepal despite adequate sunlight.

## 7. Recommendations

This study highlights the need to create public awareness regarding the importance of vitamin D for good health and the multiple adverse health consequences due to its deficiency. Awareness regarding sun exposure on bare skin for an adequate amount of time without using sunscreen should be emphasized. Urbanization and the rush of city life may not spare adequate duration in the sun, in which case nutritional sources and supplements become important. Health organizations and policymakers at regional and national levels need to issue food fortification policies as well as formulate national guidelines for initiating cost-effective regimens for treating vitamin D deficient people.

## Figures and Tables

**Table 1:** Baseline characteristics of participants according to vitamin D status.

Characteristics		Total (*N*=324)		Serum vitamin D [25(OH)D] status						*p* value
				Deficient^a^		Insufficient^b^		Sufficient^c^		
		N	%	N	%	N	%	N	%	
Age groups	18–44 y	155	47.8	88	27.2	51	15.7	16	4.9	0.1
	45–65 y	169	52.2	93	28.7	47	14.5	29	9.0	

Gender	Male	143	44.1	79	24.4	37	11.4	27	8.3	*0.04*
	Female	181	55.9	102	31.5	61	18.8	18	5.6	

Ethnicity	Brahman and Chhetri	136	42.0	79	24.4	37	11.4	20	6.2	0.6
	Newar	28	8.6	13	4	13	4	2	0.6	
	Janajati	130	40.1	73	22.5	38	11.7	19	5.9	
	Occupational caste (Dalit)	30	9.3	16	4.9	10	3.1	4	1.2	

Region of residence	Sunsari	102	31.5	52	16.0	37	11.4	13	4.0	0.2
	Morang	222	68.5	129	39.8	61	18.8	32	9.9	

Education	Up to primary level	72	22.2	31	9.6	24	7.4	17	5.2	*0.02*
	Intermediate level	127	39.2	77	23.8	32	9.9	18	5.6	
	High school	59	18.2	33	10.2	23	7.1	3	0.9	
	Above high school	66	20.4	40	12.3	19	5.9	7	2.2	

Occupation	Professional and semiprofessional	54	16.7	34	10.5	16	4.9	4	1.2	0.4
	Skilled and semi-skilled work	84	25.9	42	13	24	7.4	18	5.6	
	Arithmetic skilled jobs	42	13.0	24	7.4	13	4.0	5	1.5	
	Unskilled work	33	10.2	22	6.8	9	2.8	2	0.6	
	Unemployed	111	34.3	59	18.2	36	11.1	16	4.9	

Body mass index	Normal	147	45.4	80	24.7	46	14.2	21	6.5	0.9
	Overweight	81	25.0	47	14.5	22	6.8	12	3.7	
	Obese	96	29.6	54	16.7	30	9.3	12	3.7	

Socioeconomic status	Upper, middle, and above	83	25.6	51	15.7	26	8	6	1.9	0.3
	Lower middle	112	34.6	61	18.8	33	10.2	18	5.6	
	Lower class	129	39.8	69	21.3	39	12	21	6.5	

Smoking status	Current smoker	59	18.2	31	9.6	17	5.2	11	3.4	0.5
	Former or never smoker	265	81.8	150	46.3	81	25	34	10.5	

Alcohol intake	Drinks alcoholic beverages	110	34.0	64	19.8	34	10.5	12	3.7	0.5
	Does not drink	214	66.0	117	36.1	64	19.8	33	10.2	

Physical activities	Moderate	73	22.5	40	12.3	25	7.7	8	2.5	0.7
	Active	87	26.9	51	15.7	25	7.7	11	3.4	
	Sedentary	164	50.6	90	27.8	48	14.8	26	8	

Skin colour	Fair	92	28.4	55	17.0	27	8.3	10	3.1	***0.001***
	Light brown	143	44.1	64	19.8	57	17.6	22	6.8	
	Dark brown	89	27.5	62	19.1	14	4.3	13	4.0	

Time of maximum sun exposure	Early morning	108	33.3	59	18.2	25	7.7	24	7.4	***0.02***
	Late morning	167	51.5	93	28.7	58	17.9	16	4.9	
	Afternoon	49	15.1	29	9	15	4.6	5	1.5	

Duration of sun exposure	<15 minutes	175	54.0	112	34.6	43	13.3	20	6.2	***0.004***
	15–30 minutes	86	26.5	36	11.1	31	9.6	19	5.9	
	>30 minutes	63	19.4	33	10.2	24	7.4	6	1.9	

Sunscreen	Uses SPF 15 and above	80	24.7	41	12.7	26	8.0	13	4.0	0.6
	Does not use	244	75.3	140	43.2	72	22.2	32	9.9	

Total N% represents the number and percentage of rows. Deficient^a^: <20 ng/ml, insufficient^b^: 29 and 20 ng/ml, and sufficient^c^: 30–100 ng/ml 25(OH)D. *p* values <0.05 are significant. *p* value shown in italics was found to be significant on *χ*^2^ test. The *p* values in bold italics were found to be significant even after post hoc test using the Bonferroni correction.

**Table 2:** Dietary pattern and vitamin D status.

Characteristics		Total		Serum vitamin D [25(OH)D] status						*p* value
				Deficient		Insufficient		Sufficient		
		N	%	N	%	N	%	N	%	
Diet	Nonvegetarian	305	94.1	171	52.8	92	28.4	42	13	0.8
	Vegetarian	19	5.9	10	3.1	6	1.9	3	0.9	

Meat-type	Mostly chicken	123	38.0	53	16.4	44	13.6	26	8	***0.01***
	Chicken and mutton	109	33.6	72	22.2	26	8	11	3.4	
	Chicken and pork	68	21.0	43	13.3	19	5.9	6	1.9	
	None	24	7.4	13	4	9	2.8	2	0.6	

Frequency of meat intake	2–3 times/month	50	15.4	28	8.6	12	3.7	10	3.1	0.6
	Once a week	147	45.4	87	26.9	42	13	18	5.6	
	≥2/week	103	31.8	53	16.4	35	10.8	15	4.6	
	None	24	7.4	13	4	9	2.8	2	0.6	

Frequency of fish intake	Monthly	199	61.4	109	33.6	69	21.3	21	6.5	***0.001***
	Weekly	91	28.1	45	13.9	23	7.1	23	7.1	
	Rarely/never	34	10.5	27	8.3	6	1.9	1	0.3	

Frequency of milk intake	Less than once a week	168	51.9	101	31.2	47	14.5	20	6.2	0.3
	1–2/week	97	29.9	52	16	32	9.9	13	4	
	≥3/week	59	18.2	28	8.6	19	5.9	12	3.7	

Frequency of dairy products intake	Less than once a week	115	35.5	77	23.8	30	9.3	8	2.5	***0.02***
	1–2/week	118	36.4	60	18.5	38	11.7	20	6.2	
	≥3/week	91	28.1	44	13.6	30	9.3	17	5.2	

Total N% represents the number and percentage of rows. *p* value shown in bold italics was significant after the post hoc test using the Bonferroni correction.

**Table 3:** Multivariate analysis of vitamin D deficiency and insufficiency.

Characteristics	Vitamin D status			
	Vitamin D deficiency		Vitamin D insufficiency	
	Crude odds^1^ (95% CI)	Adjusted odds^2^ (95% CI)	Crude odds^1^ (95% CI)	Adjusted odds^2^ (95% CI)
Duration of sun exposure				
>30 minutes	0.9 (0.3–2.6)	1.2 (0.4–3.6)	1.8 (0.6–5.2)	1.8 (0.6–5.6)
15–30 minutes	0.3 (0.1–0.7)^a^	0.5 (0.2–1.1)	0.7 (0.3–1.6)	1 (0.4–2.3)
<15 minutes	1.0	1.0	1.0	1.0
Skin colour				
Fair	1.1 (0.4–2.8)	0.7 (0.3–2.1)	2.5 (0.8–7.1)	1.6 (0.5–4.9)
Light brown	0.6 (0.2–1.3)	0.5 (0.2–1.2)	2.4 (0.9–5.9)	1.8 (0.6–4.7)
Dark brown	1.0	1.0	1.0	1.0
Time of maximum sun exposure				
Early morning	0.4 (0.1–1.2)	0.5 (0.2–1.9)	0.3 (0.1–1.1)	0.5 (0.1–1.9)
Late morning	1.0 (0.3–2.9)	1.3 (0.4–4.4)	1.2 (0.3–3.8)	1.6 (0.4–5.4)
Afternoon	1.0	1.0	1.0	1.0
Frequency of dairy intake				
≥3/week	0.2 (0.1–0.6)^b^	0.3 (0.1–0.8)^c^	0.4 (0.1–1.2)	0.6 (0.1–1.6)
1–2/week	0.3 (0.1–0.7)^d^	0.33 (0.1–0.8)^e^	0.5 (0.1–1.3)	0.5 (0.1–1.4)
Less than once a week	1.0	1.0	1.0	1.0
Frequency of fish intake				
Monthly	0.1 (0.02–1.4)	0.1 (0.01–1.5)	0.5 (0.06–4.8)	0.8 (0.08–7.8)
Weekly	0.07 (0.009–0.5)^f^	0.06 (0.008–0.6)^g^	0.1 (0.01–1.4)	0.2 (0.02–2.4)
Rarely/never	1.0	1.0	1.0	1.0
Meat-type				
Mostly chicken	0.3 (0.06–1.4)	0.9 (0.1–5)	0.3 (0.07–1.8)	0.6 (0.1–3.5)
Chicken and mutton	1 (0.2–5)	3 (0.5–18)	0.5 (0.09–2.8)	1 (0.2–6)
Chicken and pork	1.1 (0.1–6)	3.6 (0.5–25)	0.7 (0.1–4)	1.4 (0.2–11)
None	1.0	1.0	1.0	1.0

OR: odds ratio, CI: confidence interval. ^1^Univariate logistic regression,^2^ multivariate logistic regression using sufficient vitamin D status as reference. ^a^*p* value: 0.004. ^b^*p* value: 0.005. ^c^*p* value: 0.02. ^d^*p* value: 0.01. ^e^*p* value: 0.02. ^f^*p* value: 0.01. ^g^*p* value: 0.01.

## Data Availability

The datasets used in this study are available from the corresponding author upon request.
